# The Role of Mifepristone in Meningiomas Management: A Systematic Review of the Literature

**DOI:** 10.1155/2015/267831

**Published:** 2015-06-03

**Authors:** Giulia Cossu, Marc Levivier, Roy Thomas Daniel, Mahmoud Messerer

**Affiliations:** ^1^Service of Neurosurgery, Department of Clinical Neuroscience, Faculty of Human Medicine and Biology, University Hospital of Lausanne, 46 rue du Bugnon, 1011 Lausanne, Switzerland; ^2^Department of Neurosurgery, University Hospital of Bicetre, Faculty of Medicine of Paris Sud, 78 rue du Général Leclerc, 94270 Le Kremlin-Bicêtre, France

## Abstract

*Objectives*. We performed a systematic literature review to analyze the clinical application and the safety of mifepristone, a prominent antiprogesterone agent, in meningioma patients. *Materials and Methods*. A systematic search was performed through Medline, Cochrane, and clinicaltrials.gov databases from 1960 to 2014. *Study Selection*. Studies were selected through a PICO approach. Population was meningioma patients, meningioma cells cultures, and animal models. Intervention was mifepristone administration. Control was placebo administration or any other drug tested. Outcomes were clinical and radiological responsiveness, safety profile, and cell growth inhibition. *Results*. A total of 7 preclinical and 6 clinical studies and one abstract were included. Encouraging results were found in preclinical studies. Concerning clinical studies, the response rate to mifepristone in terms of radiological regression and symptomatic improvement/stability in patients with inoperable meningioma was low. In meningiomatosis, favorable preliminary results were recorded. The safety profile was good. Limitations were as follows. The tumoral expression of progesterone receptors was not analyzed systematically in every study considered. *Conclusions*. No clear evidence exists to recommend mifepristone in inoperable meningiomas. Preliminary encouraging results were found in diffuse meningiomatosis. Mifepristone is a well-tolerated treatment. Patients' selection and hormonal profile analysis in meningiomas are fundamental for a better understanding of its benefit. Multicenter placebo-controlled trials are required.

## 1. Introduction

Meningiomas represent the most common primary intracranial neoplasm, with an annual incidence of 7-8 cases/100000 individuals [1]. Surgery represents the first line of treatment for symptomatic or growing meningiomas and the recurrence rate is proportional to the extent of resection [2]. Gross total resection may be challenging with lesions located near critical neurovascular structures or highly infiltrating the bone or the dura. Technical advances in microsurgical techniques, endoscopic approaches, and radiation therapies, especially radiosurgery, have recently improved the management of recurrent meningiomas.

Epidemiological studies have reported a higher prevalence of meningioma in females [1] and further studies showed a positive association between meningioma and breast cancer with a twofold increase in risk [3]. Similarly, waxing and waning of clinical manifestations during pregnancy, more evident during the last four months of gestations, were observed [4, 5]. These observations led to the hypothesis that meningioma growth may be hormonally affected or dependent.

Donnell et al. [6] first described estrogen receptor (ER) expression in meningioma specimens. Further histochemical staining has shown how, contrarily to breast cancer cells, meningioma cells present a high expression of progesterone receptors (PR) and only a weak positivity for ER [7–9]. In most progesterone receptor positive tissues, estrogens modulate the presence of PR [10] but this seems not to be the case for meningiomas. PR were found also in normal leptomeningeal tissue and they may influence the normal functioning of the meninges [11].

Many hormonal antagonists have been used for antineoplastic purpose. Mifepristone (RU-486) was a French discovery in 1982 and it has been used as a drug to terminate early pregnancy. It is an oral progesterone antagonist with a minor affinity for the glucocorticoid receptor [12]. Given its different hormonal and endocrine properties, a number of clinical trials started to investigate its alternative applications in the oncologic field [13–21]. The aim of this paper is to perform a systematic review of the literature regarding the oncological use of mifepristone in meningiomas, to clarify its clinical indications and the long-term safety.

## 2. Materials and Methods

The literature search was performed by one of the authors (Giulia Cossu) through the electronic databases Medline and Cochrane from January 1960 till December 2014, using the search terms “mifepristone” and “meningioma,” as free text. We also manually reviewed the reference lists of identified studies and scanned abstracts from recent (from 2001 to 2015) conference proceedings. Finally, we searched for ongoing trials on https://clinicaltrials.gov/.

### 2.1. Study Selection

Two authors (Giulia Cossu and Mahmoud Messerer) reviewed independently full-text articles, abstracts, and citations to select pertinent studies. Search was focused on the usefulness of mifepristone treatment and treatment related side effects in patients with meningiomas: the study selection was based on a PICO approach. Population included patients with a diagnosis of meningioma, both confirmed histologically and suspected by radiological characteristics. Intervention was mifepristone administration and the control was represented by the administration of placebo or another drug. The primary outcomes considered were the radiological response (diminution or stabilization of tumor volume assessed through serial CT or MRI) and the clinical improvement, either subjectively declared from the patients or objectively assessed through a medical examination, evaluated during and/or after treatment administration. The secondary outcomes evaluated were the clinical tolerance and the side effects related to mifepristone treatment. Randomized controlled trials were preferred but also full-text articles of retrospective or prospective studies evaluating the efficacy or the feasibility of mifepristone treatment in meningiomas were included. Preclinical studies, using cell cultures or animal models, were also considered. Considering the paucity of literature data on the topic, we decided to include case reports and case series when satisfying clinical and radiological evaluations were present. Abstract and posters reporting unpublished data were included. Review studies and studies not in English were not further considered.

In case of disagreement about the inclusion of a study, this was discussed and considered eligible only if a consensus from all the examiners was reached.

## 3. Results

With our literature search, 67 articles were identified and screened on the basis of title and abstract. Two abstracts were also considered pertinent to the topic.

**Figure 1:**
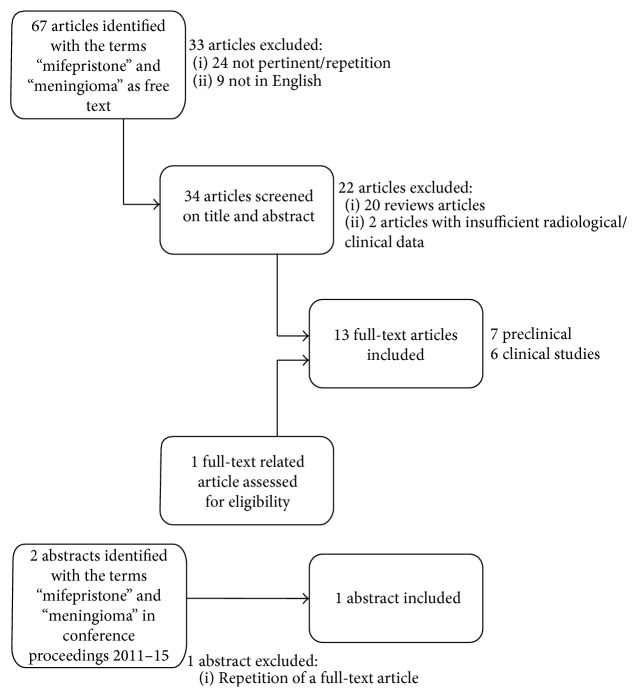
Flow chart.

A total of 7 preclinical and 6 clinical studies and one abstract were included in our systematic review (Figure 1, flow chart).

### 3.1. Preclinical Studies (Table 1)

In 1986, Olson et al. [22] first demonstrated the efficacy of mifepristone in inhibiting cell growth in three cultures of human meningioma cells after 28 days of treatment. The histological grade was not specified but all the specimens were positive for PR and weakly positive for ER. Mifepristone seemed to compete with progesterone for the progesterone-binding protein site in meningioma cells with an inhibition of cell growth ranging between 18 and 36%. A clear dose effect was seen only in one of three specimens, thus indicating that a competitive binding may not be solely responsible for these results [22].

Blankenstein et al. [23] reported similar results in 30 cultures of human meningioma cells exposed to estrogen, progesterone, tamoxifen, and mifepristone at variable concentrations for 8 days. Cell growth was significantly reduced by mifepristone exposure (*p* < 0.05) when compared to control cultures.

According to Koper et al. [24], progesterone and mifepristone may modulate the sensibility of meningioma cells to the epidermal growth factor (EGF): culture cells treated with mifepristone showed in fact a diminished response to the mitogenic effect of EGF. However, the PR expression was not analyzed in one case and the EGF receptors (EGFR) expression was not specified in 2 cases.

Schrell et al. [25] showed no effect from progesterone or mifepristone exposition in 23 meningioma cell cultures with varying degrees of PR expression. The thymidine-labeled uptake was unaffected by progestogenic treatment and the DNA polymerase activity was not correlated with the PR expression. Analogously, Wilisch-Neumann et al. [26] reported a very limited response from mifepristone treatment: the growth inhibition was evident in one of four analyzed specimens and the concentration of mifepristone needed was too elevated to be used in common clinical practice.

Matsuda et al. [27] showed interesting results: they conducted an* in vitro* study associated with an* in vivo* evaluation after implantation of tumoral cells in the subrenal capsule of nude mice. Mifepristone showed a cytostatic and a cytocidal effect independently from PR expression* in vitro*. In fact, over 9 specimens not expressing PR, 7 responded to treatment. Similar results were found in the animal model, where an inhibition of tumor growth was independent of PR expression in the meningioma cells implanted.

Olson et al. [28] used 6 nude mice to perform a placebo-controlled study. They obtained satisfying results with regression of the volume of the implanted meningioma in two of three cases, and only a small amount of cells was found in the third case receiving mifepristone.

*In vitro* and preclinical studies supported thus the realization of clinical trials to evaluate the efficacy of mifepristone in patients with unresectable meningioma.

### 3.2. Clinical Trials in Meningioma Patients (Table 2)

All included full-text studies were retrospective. Five studies evaluated the effect of mifepristone therapy on the clinical and radiological follow-up of meningioma patients [29–33]. One study focused on the evaluation of side effects of long-term mifepristone treatment [34]. All the studies considered focused on patients with unresectable or recurrent meningiomas, with progression at clinical or radiological follow-up in most of the cases, with the exception of the study of Touat et al. [33], which was conducted on three patients with diffuse meningiomatosis.

The abstract included reported preliminary results of a phase III double-blind randomized placebo-controlled study conducted in patients with unresectable meningiomas [35].

The dose of 200 mg/day was chosen to obtain a good antiprogesterone effect without a significant antiglucocorticoid activity [36, 37]. The duration of the treatment varied among the studies from 2 to 31 months, and the follow-up period extended from the duration of the treatment to 14 years. To avoid adrenal insufficiency most authors introduced a substitutive treatment of dexamethasone during the first weeks or during the whole length of the treatment [29–32].

#### 3.2.1. Therapeutic Effects

Grunberg et al. reported preliminary results in 14 patients in 1991 [29]. Thirteen patients were evaluated for the clinical and radiological follow-up and in 5 of the 13 patients (38%) tumor regression was recorded, accompanied by a subjective symptomatic improvement in 3 of them (23%). Three patients experienced progression (23%) and two of them had malignant meningioma. Daily oral mifepristone for a period of 2 years or more was well tolerated.

Given these encouraging results, the same group started a phase III randomized double-blind placebo-controlled trial on the usefulness of mifepristone treatment on 160 patients with progressive meningiomas. An abstract was presented in 2001 and authors failed to demonstrate improvement in freedom from progression in mifepristone group (*p* = 0.44) [35]. However hormone receptors expression was not specified. These results were never further published.

Grunberg et al. reported in 2006 more detailed results in 28 patients, including the patients previously enrolled in the study of 1991 [30]. The mean follow-up was 35 months. A radiological improvement with reduction in tumor size was reported in 5 of the 28 patients (17%) and a clinical improvement was evident in 3 patients out of 28 (11%). Seven of 8 patients experimenting regression (both clinical and radiological) were males or premenopausal females.

Lamberts et al. [31] evaluated a cohort of 10 patients with 12 recurrent or inoperable meningiomas, progressing at follow-up. They recorded a transient regression in 33% of the tumors (4/12) with further radiological progression after the discontinuation of the treatment. The disease was stable in 25% cases (3/12), while 42% (5/12) progressed. Fifty percent of the patients (5/10) reported clinical improvement, objectively assessed in 2 cases. No data about PR expression were provided.

De Keizer and Smit [32] reported a case series of 2 patients treated with long-term mifepristone for unresectable sphenoid-ridge meningioma, with a follow-up of 14 years. Both patients experienced radiological stability and symptomatic improvement: one reported an improvement in visual acuity and the other experienced regression of his headache with no changes in visual acuity. Both patients progressed and deteriorated after treatment discontinuation with a new stabilization after the restarting of the treatment.

Touat et al. [33] enrolled three postmenopausal patients with diffuse meningiomatosis. The histological examination was performed only in one case. All the patients experimented long-lasting clinical remission and in two of them meningiomas stabilized or diminished in size after mifepristone treatment.

#### 3.2.2. Side Effects

The most common described side effect of mifepristone therapy is asthenia. Severe fatigue however is rare. Some patients experienced anorexia, vomiting, and nausea [29, 34]. All these symptoms may be attributed to the blockade of glucocorticoid receptors and an improvement of these manifestations with an exogenous corticosteroid treatment was described [29, 31]. The majority of the studies supplemented patients under mifepristone treatment with daily low-dose of dexamethasone, at least for the first two weeks or for the whole duration of the treatment.

Hot flashes, gynecomastia or breast tenderness, decreased libido, and cutaneous rash were also observed [29]. The cessation of menses was an expected side effect in premenopausal patients, with a return to a normal menstrual cycle after a variable period of time after the discontinuation of the treatment [29]. Supplementation in testosterone helped in normalizing libido in male patients [29]. Mifepristone treatment was associated with the development of endometrial hyperplasia [30, 32, 34, 35].

One case of peritoneal adenocarcinoma [30] and one case of benign ovarian serous cystadenoma [33] were reported. The association with mifepristone treatment is unknown.

Subclinical hypothyroidism was observed [38] and some authors reported transient elevation of hepatic enzymes during mifepristone treatment for other conditions [39, 40] but this finding was not confirmed by Spitz et al. in meningioma patients [34].

## 4. Discussion

Meningiomas have a significant association with hormone-dependent conditions [3, 4, 41] and since the discovery of the high prevalence of PR expression in meningiomas, the option to use hormonal therapy as cytostatic agent became a real therapeutic strategy.

Mifepristone is a 19-norsteroid agent presenting a high affinity for the PR and a lower but considerable affinity for the glucocorticoid receptor [12]. The abortifacient properties of mifepristone were early discovered and the use of this drug is currently approved for early termination of pregnancy, cervical dilatation for surgical abortion, and management of early embryonic loss [42]. Further applications to the endocrine and oncological fields remain under investigation.

The expression of PR in meningioma cells was early characterized in 1979 [6] and, since then, different hormonal therapies have been used in preclinical studies and clinical trials. Most of them showed contrasting results [43–48], while initial encouraging results were found for mifepristone in* in vitro* studies and animal models.

Clinical studies on the efficacy of the use of mifepristone in meningiomas showed contrasting results. Grunberg et al. [29] in their study of 1991 reported positive results in 38% patients with inoperable meningiomas. A further enrollment allowed the same group to publish more extensive data: in 2006, the authors failed to show new cases of radiological or clinical response to mifepristone treatment [30]. Analogously in 2001 they presented in an abstract the results of a placebo-controlled RCT in patients with unresectable meningiomas: no significant difference on freedom from progression was observed between the two arms [35]. Unfortunately, limited data are available for this trial.

Lamberts et al. [31] showed a stable disease or a transient regression in tumor volume in 58% of cases (7/12 meningiomas), but when the time course of tumor size (volumetric data) was analyzed in more details, the results were no more convincing. The four tumors decreasing in size showed a progressive reduction of tumor volume only in one case during mifepristone treatment, while in two cases a transient precocious regression was followed by a dimensional increase during mifepristone treatment. In one case, an initial stability was followed by a slight reduction of tumor volume at one year of treatment.

De Keizer and Smit [32] showed a radiological stability and a symptomatic improvement in both patients studied, having an unresectable sphenoid-ridge meningioma.

Two case reports, not included in the primary analysis because of the lacking of precise radiological data, confirmed a radiological and clinical stability with long-term mifepristone treatment in two patients [49, 50] and a decrease in tumor size in one case [50].

Long-term treatment with mifepristone is generally well tolerated. Adrenal insufficiency may be seen with very high doses of mifepristone, where the compensatory elevation of endogenous cortisol becomes insufficient, but in most of cases the introduction of a low-dose therapy of daily dexamethasone* per os* was sufficient to relieve the mild symptoms of corticotropic insufficiency (asthenia, nausea, and vomiting) [30, 31].

Mifepristone treatment was associated with the development of endometrial hyperplasia [30, 32, 34] and endometrial polyps [49]. The hormonal pathways determining this phenomenon are still unknown. Heikinheimoab et al. [38] investigated the effects of long-term mifepristone treatment (200 mg/d for at least 15 months) on sex steroids and gonadotrophins levels. A prolonged treatment is associated with increased serum levels of androstenedione, testosterone, estrone, and estradiol, probably through the inhibition of glucocorticoid receptors. An increase in cortisol level secondary to mifepristone administration may be in fact associated with an increase in adrenal production of androgens with a secondary aromatization to estrogens [38, 51, 52]. The proliferative effects of estrogens are in these cases no more counteracted by progesterone.

The case of subclinical hypothyroidism recorded [38] was probably linked to an interaction with the corticotropic axis [53, 54] or due to an effect on synthesis and/or release of thyroxine (biologically characterized by an elevation of thyroid-stimulating hormone and a decrease in thyroxin) rather than a direct interaction of mifepristone with the thyroxine receptor [55].

In conclusion, mifepristone seems to act on the progesterone, glucocorticoid, androgen, and thyroid pathways on the long term.

From our literature analysis only retrospective studies were identified and included as full-text articles. One RCT was only available as abstract. Thus, no class I evidence may be extrapolated. Also two case series, respectively, of 2 [32] and 3 patients [33] were included. The experience in clinical practice is very limited and the major evidence derives from one single center [29, 30, 35].

The studies were extremely heterogeneous both in the preclinical and in the clinical groups, in terms of population included, duration of treatment, and length of follow-up. For all these reasons, a meta-analysis was not performed.

In clinical studies, a heterogeneous cohort of patients in terms of sex, menopausal status, histology, and expression of PR was enrolled and data were pooled together in the majority of cases. Furthermore, some authors included cases with no histologic diagnosis [29, 30] and the specific expression of PR was not analyzed in most of cases [29–31, 33].

The selection of the population of interest may be determinant: Grunberg et al. [30] observed significant responses in males and in premenopausal women, and further studies focusing on this population may help in detecting clinically significant responses deriving form mifepristone treatment.

The histology and the WHO grade may be important in determining the response to mifepristone treatment. A lower presence of PR was noted in anaplastic meningiomas [56] and PR expression was inversely correlated with Ki67 expression and WHO grading [57, 58]. Thus, mifepristone may not be a good treatment for anaplastic and malignant meningiomas.

Encouraging results support the use of mifepristone in a clinical trial for patients with diffuse meningiomatosis. Touat et al. [33] enrolled three patients and all showed a long-lasting clinical improvement during the treatment, associated with two cases with a radiological shrinking or stabilization. From epidemiological studies, it is evident that diffuse meningiomatosis is characterized by a more pronounced female predominance and immunohistochemical studies showed a higher PR expression in this subgroup of meningiomas [59].

Progesterone receptors exist as two isoforms (PRA and PRB) codified from the same gene by different promoters [60]. Increasing literature evidence shows how they have different functions [61, 62] and PRA seems to be downregulated in models of antiprogestin resistant breast cancers [63, 64]. Mifepristone showed a major action in preclinical models of breast cancers overexpressing PRA when compared to cancers having a high PRB expression [65]. These effects may be useful in guiding the choice of target patients and it would be interesting to analyze more in details the relationship between the PR isoforms expressed and the responsiveness to mifepristone treatment in meningioma patients.

Some authors support the hypothesis that the antitumoral effect of mifepristone may not depend directly on the expression of PR [66]. Koper et al. [24] showed how progesterone may act* in vitro* through the modulation of the cellular response to various mitogenic stimuli as EGF, insulin, and insulin-like growth factor 1 (IGF-1). Mifepristone inhibits growth induced by EGF* in vitro* [24] and it may block the activation of IGF-1 signaling in some breast cancer cell lines [67]. These may be additional mechanisms of action of this drug. Furthermore, mifepristone inhibited cell migration, abnormal adhesion to basement membrane, and angiogenesis in an animal model of adenocarcinoma [68]. Thereafter, it was shown to promote apoptosis through the activation of the pathway of caspase-3 [18]. Further investigations should be conducted to study the interactions between mifepristone and its modulations of signal transduction pathways.

Further limitations of the studies included in this review are related to the volumetric assessment of tumor size, which was not recorded systematically by all the authors, and the radiological techniques varied between CT scan and MRI, even in the same studies. Indeed, a well-performed MRI is more sensible in detecting minimal changes in size than a simple CT evaluation. Also, the length of the treatment and the follow-up period should be sufficiently long to detect minimal volumetric changes; the effects of a hormonal therapy may in fact be evident after a certain period of time, because of the slow proliferation rate of meningioma cells. Furthermore, clinical improvement should be objectively documented when possible (periodical neurological examination of cranial nerve palsies, ophthalmological evaluation for visual acuity and visual fields, etc.).

Through the analysis of the literature, only minor evidences exist to recommend mifepristone treatment in surgically inoperable progressing meningiomas. This hormonal therapy has a safe profile and a good clinical tolerance, but the clinical benefit is very limited and it should be recommended only in an experimental setting. The indications for diffuse meningiomatosis are still preliminary. Well-designed multicentered placebo-controlled trails with a long follow-up should be realized to finally determine the real value of mifepristone treatment.

Mifepristone may represent a starting point towards new perspectives: other hormonal therapies and in particular other progesterone antagonists with higher affinity for PR may represent a real future alternative. Furthermore advances in molecular and genomic studies are actually targeting the molecular changes in tumorigenic mechanisms to obtain more selective therapies. Inhibitors of EGFR or PDGFR may inhibit meningioma growth but preliminary studies failed to show a clinical benefit [69, 70]. Less selective tyrosine kinase receptor inhibitors, like sunitinib, may be more promising [70, 71]. Also antiangiogenetic molecules may represent a valuable option [72].

## 5. Conclusions

The recurrence rate is high for meningiomas incompletely resected and different therapeutic options were explored for the management of recurrent or inoperable meningiomas. The characterization of hormonal receptors, in particular PR, opened new ways towards antiprogestogens as therapeutic strategies.

According to literature analysis no clear evidence exists to recommend mifepristone treatment in recurrent and inoperable meningiomas. A possible application may be found in diffuse meningiomatosis but further confirmations are needed. Mifepristone is a well-tolerated treatment; however a preliminary selection of the patients is fundamental.

It is time the clinical experience available from single center studies is substituted by well-designed multicenter placebo-controlled studies.

## Figures and Tables

**Table 1:** 

Study	Year	*N*° specimens/animals	Purpose	Population	Histology/PR expression	Intervention	Outcome and results	Conclusion
Olson et al. [22]	1986	3	Evaluation of hormonal stimulation and hormonal deprivation	Human meningioma cell cultures	Histology ns ER and PR positive	Exposition to 10^−6^–10^−10^ M mifepristone for 28 d	Mifepristone inhibits cell growth (18–36%) in all 3 specimens when compared to the control medium (Coulter counter)	PRO

Blankenstein et al. [23]	1989	30 cultures form 39 tissue specimens	Evaluation of mifepristone as inhibitor of cell growth	Human meningioma cell cultures	PR expression measured but ns in the text Histology ns	Exposition to 10^−6^–10^−10^ M mifepristone for 8 d	Growth inhibition with mifepristone exposure (decrease in TLI, thymidine labeling index *p* < 0.05)	PRO

Koper et al. [24]	1990	6	Effect of progesterone and mifepristone on the response to EGF	Human meningioma cell cultures 2 M	PR expression ns in 1 case EGFR expression ns in 2 cases	3 d incubation with 10^−6^ M mifepristone	EGF-stimulated incorporation of thymidine was reduced with mifepristone exposure	PRO

Schrell et al. [25]	1989	23	Evaluation of effects of progesterone/mifepristone on cell growth	Human meningioma cell cultures	Varying degrees of PR expression	Exposition to 10^−6^–10^−10^ M mifepristone for 6–12 d	No stimulating nor inhibitory effect was found	CON

Wilisch-Neumann et al. [26]	2014	4 (plus 3 other cell lines NF2wt and NF2ko)	Efficacy of different chemotherapies/target therapies on meningioma cells growth	Human meningioma cell lines: *in vitro* analysis	2 benign and 2 malignant meningiomas; PR expression ns	Exposition to different mifepristone concentration, till 10^−6^ M	Mifepristone showed some efficacy in growth inhibition only at very high doses and only in one cell line	CON

Matsuda et al. [27]	1994	13 meningioma cell cultures 51 nude mice, female athymic, divided into 3 groups	Antitumor effects of mifepristone and onapristone and relationship with PR expression	Human meningioma tissue (13 specimens), previously untreated Cell cultures and cells implanted in subrenal capsule of athymic mice	9 meningothelial 2 transitional 1 angiomatous 1 fibroblastic 4/13 PR + 3 tumors implanted 2 meningothelial PR+ 1 angiomatous PR−	Exposition to 10^−6^–10^−10^ M of antiprogestogen *in vitro*, evaluated at 9 d and 21 d Injection 0.4 mL of mifepristone, onapristone, or placebo for 28 d	Antitumor effects via PRs and/or another receptor *In vitro:* cytostatic and cytocidal effects regardless of the presence of PRs (7/9 PR- responded) *In vivo:* reduction of tumor volume regardless of the presence of PR (no necrosis)	PRO

Olson et al. [28]	1987	6 nude mice, male athymic, divided into 2 groups	Effect of mifepristone on meningioma implanted into nude mice	1 human meningioma implanted in the subcutaneous tissue of nude mice	Meningothelial histology PR expression	Mifepristone 10 mg/kg/d for 3 mo	Disappearance of implanted meningioma nodules in 2/3 mice in 8–10 w; a small number of cells in the third case	PRO

CON: against; d: days; EGF: epithelial growth factor; ER: estrogen receptors; M: molar; ns: not specified; PR: progesterone receptors; PRO: for, in favor of; w: weeks.

**Table 2:** 

Study	Year	*N*° pts	Purpose	Population	Histology/PR expression	Intervention	Outcome and results	Side effects	Limitations	Conclusion
Grunberg et al. [29]	1991	14	Efficacy of long-term oral therapy with mifepristone in unresectable meningiomas 11 pts were progressing (radiologically or symptomatically) before starting the TT with mifepristone	Inoperable meningiomas 6 M 8 F: (i) 2 premenop (ii) 6 postmenop	5 meningothelial 3 cellular 2 fibrous meningioma 2 malignant meningioma 2 w/o biopsy No evaluation of PR expression	Mifepristone 200 mg/d for 2–31 mo (9 pts more than 12 mo) Dexa 1 mg daily during the first 14 d of treatment	13 pts assessed for response (1 refused) (i) 5/13 pts radiological regression after at least 6 mo (ii) 3/13 subjective clinical improvement after 2-3 mo (iii) 3/13 progression (of which 2 were malignant meningiomas)	14 evaluated (i) 11/14 fatigue (ii) 5/14 hot flashes (iii) 3/6 M gynecomastia (iv) 2 alopecia (v) 2 amenorrhea (premenop F) (vi) 1 hypothyroidism	Subjective clinical evaluation Radiological response evaluated with CT or MRI Heterogenous population: (i) 2 premenop F (ii) 6 postmenop F (iii) 6 men No evaluation of PR expression 1 previous RT 4 previous hormonal therapy (MPA and tamoxifen) For the 3 pts defined as stable before starting mifepristone, the previous FU is ns	PRO

Grunberg et al. [30]	2006	28 (Included the 14 pts of the study of Grunberg et al. 1991 [29])	Clinical tolerance of long-term treatment with mifepristone	9 M 19 F: (i) 5 premenop (ii) 14 postmenop	13 meningothelial 4 fibroblastic 5 cellular/ns 2 malignant 4 not biopsied No evaluation of PR expression	Mifepristone 200 mg/d for 2–31 mo Mean FU 35 mo (2–157 mo) Total of 1626 patient-months Dexa 1 mg daily during the first 14 d of treatment	8/28 pts radiological (5) and clinical (3) improvement (7/8 were male or premenopausal F)	22/28 pts mild fatigue 13/28 pts hot flashes 6/28 pts gynecomastia 3/28 depression 1 N/V 3/28 pts endometrial hyperplasia 1 pt peritoneal adenocarcinoma	2 malignant meningiomas 4 no histological diagnosis No evaluation of PR expression	CON

Lamberts et al. [31]	1992	10	Effect of mifepristone treatment in recurrent or inoperable meningiomas recent evidence of growing: neuroradiological in 9 cases ophthalmological in 4 cases	12 recurrent or inoperable meningiomas in 10 pts 7 pts previous operation 3 males 1 premenop F (with NF2) 6 postmenop F	No data about histology nor PR expression	Mifepristone 200 mg/d for 12 mo 4 cases started Dexa 7.5 mg daily and continued throughout the study	5/12 meningiomas grew (2 deaths) 3/12 stable 4/12 transient regression (progression at discontinuation of TT) 5/10 pts symptomatic improvement objective assessment in 2 pts 1 pt continued to deteriorate	Asthenia, N/V	CT scan evaluation (planimetry) Group of aggressive meningiomas (recent evidence of growing) No data about histology nor PR expression Graphic over responsive patients Analyzable in different ways	PRO

De Keizer and Smit [32]	2004	2	Efficacy of long-term mifepristone treatment	Unresectable sphenoid-ridge meningioma 2 F: 1 postmenop F	Both tumors were PR +	Mifepristone 200 mg/d then increased to 400 mg/d Dexa 0.5 mg three times daily FU 14 y	2 controlled tumor growth 1 improved VA 1 improved headache, stable VA clinical deterioration, and radiological growth after TT discontinuation Initial benefits persist	1 endometrial hyperplasia	CT and MRI evaluation	PRO

Touat et al. [33]	2014	3	Efficacy of mifepristone in meningiomatosis	F, postmenop	Histological examination in one case	Mifepristone 200 mg/d. FU 4–10 y	3/3 long lasting clinical and 2/3 radiological response or stabilization	1 benign ovarian serous cystadenoma	Small *n*° pts Histological examination just in one case	PRO

Spitz et al. [34]	2005	25	To determine clinical side effects and biochemical abnormalities with long-term mifepristone therapy	16 F and 9 M 22–80 y unresectable meningioma		Mifepristone 200 mg/d (follow-up variable from 13 y to 4 mo) Total of 1620 mo	Safety of long-term TT	(i) 22/25 pts fatigue (ii) 2 endometrial hyperplasia (iii) 2 endometrial thickening	Varied follow-up Heterogeneous cohort: 5 premenopausal F, 11 postmenopausal F, and 9 M	PRO

Grunberg et al. [35]	2001	160 (80 per arm)	Efficacy of mifepristone in unresectable meningiomas	Adults with unresectable histologically confirmed nonmalignant meningiomas appeared (22%) or progressing (78%) within 2 y Median age 57 y 30% M 19% premenop F 51% postmenop F 29% prior RTH	ns	Blinded administration of mifepristone 200 mg/d versus placebo Follow-up 2 y	FFP (freedom from progression) Progression defined as anatomic growth or neurologic deterioration No significant difference in response between the arms: median FFP was 10 mo for mifepristone pts and 12 mo for placebo pts (*p* = 0.44)	Fatigue (72% mifepristone pts vs 54% placebo pts) Headache Hot flashes 9 mifepristone pts had endometrial hyperplasia (16% F)	Limited data available (abstract) No information about histological type and PR expression	CON

CON: against; d: days; dexa: dexamethasone; EGF: epithelial growth factor; ER: estrogen receptors; F: female; M: male; mo: months; *N*°: number; ns: not specified; N/V: nausea and vomiting; PR: progesterone receptors; Postmenop: postmenopausal; Premenop: premenopausal; PRO: for, in favor of; Pts: patients; TT: treatment; VA: visual acuity; y: years; w: weeks; w/o: without.
